# Monitoring HIV-Related Laws and Policies: Lessons for AIDS and Global Health in Agenda 2030

**DOI:** 10.1007/s10461-016-1286-0

**Published:** 2017-01-13

**Authors:** Mary Ann Torres, Sofia Gruskin, Kent Buse, Taavi Erkkola, Victoria Bendaud, Tobias Alfvén

**Affiliations:** 1 ICASO, 120 Carlton St., Suite 311, Toronto, ON M5A 4K2 Canada; 2 Program on Global Health and Human Rights, Institute for Global Health University of Southern California, Los Angeles, CA USA; 3 UNAIDS, 20 Avenue Appia, Geneva, Switzerland; 4 Global Health – Health Systems and Policy, Department of Public Health Sciences, Karolinska Institutet, Stockholm, Sweden

**Keywords:** HIV, Human rights, Policy, Monitoring and evaluation, National Commitments and Policy Instrument (NCPI)

## Abstract

The National Commitments and Policy Instrument (NCPI) has been used to monitor AIDS-related laws and policies for over 10 years. What can be learnt from this process? Analyses draw on NCPI questionnaires, NCPI responses, the UNAIDS Law Database, survey data and responses to a 2014 survey on the NCPI. The NCPI provides the first and only systematic data on country self-reported national HIV laws and policies. High NCPI reporting rates and survey responses suggest the majority of countries consider the process relevant. Combined civil society and government engagement and reporting is integral to the NCPI. NCPI experience demonstrates its importance in describing the political and legal environment for the HIV response, for programmatic reviews and to stimulate dialogue among stakeholders, but there is a need for updating and in some instances to complement results with more objective quantitative data. We identify five areas that need to be updated in the next iteration of the NCPI and argue that the NCPI approach is relevant to participatory monitoring of targets in the health and other goals of the UN 2030 Agenda for Sustainable Development.

## Resumen

El Instrumento de Políticas y Compromisos Nacionales (NCPI por sus siglas en inglés o ICPN) ha sido utilizado para monitorear leyes y políticas relacionadas con el SIDA durante más de 10 años. ¿Qué se puede aprender de este proceso? Análisis presentados están basados en los cuestionarios del ICPN, y las respuestas al mismo, la base de datos de leyes de ONUSIDA, datos de encuestas y respuestas a una encuesta sobre el ICPN llevada a cabo en el 2014. El ICPN brinda la primera y única recopilación sistemática de datos auto-reportados sobre leyes y políticas nacionales relacionadas al VIH. Altas tasas de reporte del ICPN y respuestas a la encuesta sugieren que la mayoría de los países consideran el proceso relevante. La participación y el reporte conjunto por parte de gobiernos y sociedad civil es clave para el ICPN. La experiencia del ICPN demuestra su importancia en describir el ambiente político y legal de la respuesta al VIH, para revisiones programáticas y para estimular el diálogo entre contrapartes, pero hay necesidad de actualizar la herramienta y en algunos casos complementar los resultados con datos cuantitativos objetivos. Identificamos cinco áreas que deben ser actualizadas en la próxima iteración del ICPN y argumentamos que el enfoque del ICPN es relevante para el monitoreo participativo de las metas de los objetivos de salud y otros objetivos de la Agenda 2030 para el Desarrollo Sostenible.

## Introduction

An enabling policy environment which ensures human rights and facilitates access to HIV prevention, treatment and care services has been identified as central to an effective AIDS response [1]. Monitoring of laws, policies and regulations is key to understanding how structural factors impact people’s risk of acquiring HIV, as well as their access to, and use of, services. Such monitoring can provide an assessment of how policy and legal barriers and gaps should be addressed, as well as measure progress towards more enabling policy and legal environments [2]. National AIDS programs should be guided by evidence-informed policies, and these should be based on programmatic gap analyses, accompanied by political mapping relevant to legal and policy reform [3].

Resolution 1994/24 [4] of the UN Economic and Social Council which established UNAIDS, also gave the organization the mandate to support countries to monitor their responses to AIDS. Through the United Nations General Assembly Special Session (UNGASS) 2001 Declaration of Commitment, countries agreed to conduct periodic reviews of progress towards their commitments, with civil society involvement, develop monitoring and evaluation mechanisms, and “by 2003, establish or strengthen effective monitoring systems… for the promotion and protection of human rights of people living with HIV/AIDS.” [1]. In 2002, UNAIDS, in collaboration with National AIDS Committees, civil society, academia and other partners, developed a set of indicators, including the National Composite Policy Index (NCPI), to measure progress. The 2006, 2011 and 2016 Political Declarations on HIV/AIDS [5–7] reaffirmed UNAIDS’ mandate to support monitoring and reporting on commitments.

The NCPI has been reported every two years since 2003. While keeping the same acronym, the tool’s name changed in 2012 from National Composite Policy Index to National Commitments and Policy Instrument. The NCPI is a component of the Global AIDS Response Progress Reporting (GARPR, previously UNGASS reporting). The Instrument consists of two parts: Part A is completed by governments; and Part B by non-governmental respondents including civil society, the private sector, bilateral agencies, and United Nations organizations. There is deliberate duplication of some questions between the two parts for comparison, and to encourage and facilitate country level dialogue. UNAIDS recommends that the questionnaire be completed by conducting a desk review and interviews with knowledgeable persons, validating the data through workshops with representative stakeholders and, to the extent possible, generating consensus on responses. The government focal point for the reporting process submits the completed NCPI to UNAIDS [8].

Although the focus of the NCPI is on laws, policies and regulations, over time it has evolved to also capture information on programs and their perceived implementation so that this information can be triangulated with program indicator data to facilitate dialogue and change as needed.

This paper reviews the purpose of the Instrument, the types of information which it can meaningfully capture and the value of the process for country dialogue on policy issues. This paper assesses levels of NCPI reporting and data use between 2003 and 2014 and suggests lessons and recommendations that may inform monitoring of relevant policies and legal aspects of the AIDS response and the 2030 Agenda for Sustainable Development [9] more generally.

## Methods

Analyses for this article draw on the following sources: (1) the NCPI questionnaires used in the 2004, 2006, 2008, 2010, 2012 and 2014 reporting rounds [8, 10–14]; (2) NCPI responses to Parts A and B for 2006, 2008, 2010, 2012 and 2014; (3) the UNAIDS Law Database; (4) program data on coverage of HIV testing as reported through GARPR; (5) responses to a survey conducted in 2014 as part of a review of the NCPI; (6) as well as a desk review of previous NCPI analyses.

This article is structured around three themes: (a) the purpose(s) of such an instrument; (b) the types of information which can be assessed through such an instrument; and (c) the value that its processes add at national and global levels.

To assess issues related to the purpose of the NCPI, a historical analysis of changes to the questionnaire structure and content was conducted through a desk review of NCPI questionnaires and previous analyses. Survey responses regarding the purpose of the NCPI in the context of Agenda 2030 were analyzed.

Issues around the types of information that can be meaningfully captured through the NCPI were studied in several ways. The first involved a comparison of 2014 responses to NCPI Parts A and B [8] questions on the existence of laws that present obstacles to effective prevention, treatment, care and support with available data from the UNAIDS Law Database^1^ [15, 16], to assess the reliability of the NCPI. Further responses in 2014 to questions on the extent of implementation of HIV testing and counseling in Parts A and B were compared with available program data on testing coverage among the general population and among men who have sex with men, sex workers and people who inject drugs [17] to assess reliability of NCPI data on program implementation, and on the utility of joint analysis of NCPI and program data. Testing was selected as the example as program coverage data is readily available.

The value of the NCPI reporting process at national and global level was assessed through an analysis of response rates for each reporting round, a comparative analysis of responses to Parts A and B, and survey responses related to the NCPI reporting process.

Further comparative analyses were done using responses in 2014 to questions within the Instrument on the existence of non-discrimination laws for populations most affected by the epidemic and responses in 2014 on the existence of laws that present obstacles to effective prevention, treatment, care and support from Parts A and B to identify similarities and differences and assess the value added of including similar questions in both sections of the Instrument.

A survey was conducted in 2014 as part of a review of the NCPI^2^ which collected the views of 280 respondents, including national authorities, civil society representatives, bilateral organizations, donors and United Nations organizations on the utility of the NCPI and recommendations regarding policy monitoring in the sustainable development goals (SDG) environment. The survey was disseminated to GARPR country focal points and through UN and civil society email lists, asking people to share the survey link with their networks. The survey included questions on whether the NCPI fulfilled its purpose, the applicability and relevance of such a tool for monitoring laws, polices and strategies related to the AIDS response in the Agenda 2030 context, and use of the data and findings from NCPI responses.

## Results

### Purpose of the NCPI

The purpose of the NCPI has evolved since 2004, when it only asked about the existence of national-level AIDS policies and strategies [10]. The 2006 version integrated questions from an AIDS program ‘effort’ survey to measure “the strength of effort for program inputs and outputs” to complement data from programmatic indicators [11]. Measuring progress in development and implementation of HIV laws and policies was included in the purpose statement of the NCPI from 2010 [13], although some relevant questions on law were already included in previous reporting rounds. Guidance on how to construct or calculate an index from questionnaire responses was not included in any iteration of the tool.

The NCPI’s structure has evolved to reflect these changes. The first iteration was a four section questionnaire completed by national government with inputs from other partners, including civil society. Since 2006 the NCPI included two distinct sections, Parts A and B, completed by government and non-governmental partners, respectively. The number of sub-sections increased with each reporting round to 2010 to reflect new programmatic guidance, and has since remained stable (Table 1). Since 2010, the questionnaire has included 334 questions in Part A and 166 in Part B (counting each sub-question as a separate question).

**Table 1:** Evolution of the NCPI’s structure

One questionnaire filled out by national government with inputs from other partners, including civil society	Two questionnaires (Part A and Part B) filled out by national government and non-governmental partners, respectively				
2004	2006	2008	2010	2012	2014
	Part A	Part A	Part A	Part A	Part A
Strategic plan	Strategic plan	Strategic plan	Strategic plan	Strategic plan	Strategic plan
Prevention	Political support	Political support	Political support	Political support and leadership	Political support and leadership
Human Rights	Prevention	Prevention	Prevention	Human Rights	Human Rights
Care and support	Care and support	Treatment, care and support	Treatment, care and support	Prevention	Prevention
	Monitoring and evaluation	Monitoring and evaluation	Monitoring and evaluation	Treatment, care and support	Treatment, care and support
				Monitoring and evaluation	Monitoring and evaluation
	Part B	Part B	Part B	Part B	Part B
	Human Rights	Human Rights	Human Rights	Civil society involvement	Civil society involvement
	Civil society involvement	Civil society involvement	Civil society involvement	Political support and leadership	Political support and leadership
	Prevention	Prevention	Prevention	Human Rights	Human Rights
	Care and support	Treatment, care and support	Treatment, care and support	Prevention	Prevention
				Treatment, care and support	Treatment, care and support

The NCPI has in each reporting round been officially translated from English to French, Russian and Spanish, and in some countries also translated by the UNAIDS country office (e.g. to Vietnamese in Vietnam).

Through the 2014 NCPI survey, respondents indicated potential purposes of a future iteration of the NCPI. Respondents’ proposals are consistent with its current purpose: provide a platform for dialogue among partners, in particular on the enabling environment for the HIV response; empower groups of affected populations to engage in the response; provide a snapshot of national HIV-related policies, human rights and gender issues; and assess progress and help identify challenges and areas for improvement to inform programming and advocacy.

When asked about the topics to be included in a future iteration of the NCPI, the 2014 survey respondents reiterated many issues already captured by the tool: strategic planning; policies and legal environment; political leadership and commitment, as through budget allocation; prevention, including testing and a focus on key populations; treatment, care and support; human rights, including stigma and discrimination; civil society engagement; and monitoring and evaluation. New topics were also proposed including integration, private sector engagement, subnational progress, and assessing the role of multilateral and bilateral partners from the perspectives of government and civil society. The respondents also frequently noted a need for greater attention to implementation rather than simply the existence of policy.

### Information That Can be Meaningfully Captured Through the NCPI

Even as the NCPI provides a space for regularly reporting on elements of the HIV response that may not be easily captured through other reporting mechanisms, it is important to assess what can meaningfully be included in such a questionnaire and how the results can and should be interpreted. Such an assessment must also take into account the increased availability of data around HIV-related laws and policies since the NCPI’s inception.

#### Comparison of Data on Laws Reported Through the NCPI and Available Data from Other Sources

A comparison of responses to Parts A and B of the NCPI in 2014 on the existence of laws that present obstacles to access and use of services for men who have sex with men and sex workers with data available from the UNAIDS Law Database provides insight on the reliability of the data collected through the NCPI and through other processes, and the need to review different sources in tandem to the extent possible. These two population groups were selected for this analysis due to data availability in the Law Database. Responses from government and non-governmental partners in the NCPI and data available through the UNAIDS Law Database differed between regions and for the different populations (Table 2). In most regions, fewer countries reported the existence of laws that present obstacles for men who have sex with men and sex workers through the NCPI than what is recorded in the UNAIDS Law Database, the biggest difference in this direction can be seen in West and Central Africa. The exception is Latin America where the UNAIDS Law Database recorded no country as having obstacle laws but in the NCPI both governments and civil society (to a greater extent) report the existence of obstacle laws. It should be noted that questions included in the Law Database are more specific than those included in the NCPI.

**Table 2:** Comparison of responses to Parts A and B on the existence of laws that present obstacles to effective HIV prevention, treatment, care and support in 2014 NCPI reporting round (2014) and data from the UNAIDS law database

	Number of countries reporting existence of obstacle laws NCPI Part A	Number of countries reporting existence of obstacle laws NCPI Part B	Number of countries with obstacle laws as per the UNAIDS Law Database	Total countries reporting NCPI in region
Men who have sex with men				
Asia and the Pacific	10	12	14	23
Caribbean	6	8	9	13
Eastern and Southern Africa	8	11	14	17
Eastern Europe and Central Asia	2	1	1	10
Latin America	1	5	0	15
Middle East and North Africa	11	9	12	15
West and Central Africa	7	8	13	24
Sex workers				
Asia and the Pacific	14	15	16	23
Caribbean	6	8	10	13
Eastern and Southern Africa	9	13	11	17
Eastern Europe and Central Asia	3	3	8	10
Latin America	4	8	0	15
Middle East and North Africa	10	9	14	15
West and Central Africa	7	6	13	24

Hindering or obstacle laws considered in this analysis from the UNAIDS Law Database are: sex workers—criminalization of sex work; men who have sex with men—laws against advocacy and punitive laws. The question in the NCPI 2014 reads: “Does the country have laws, regulations or policies that present obstacles to effective HIV prevention, treatment, care and support for key populations and other vulnerable subpopulations”. Responses are provided for individual population groups listed as part of the question

#### Information on Program Implementation for Triangulation with Program Data

The NCPI includes questions on the extent to which programmatic interventions, including testing and counseling, are implemented. The median implementation score reported through both NCPI Parts A and B were analyzed jointly with programmatic data on coverage of HIV testing among the general population as well as among men who have sex with men, sex workers and people who inject drugs. In the majority of regions, the median score for whether respondents considered that the majority of people in need had access to testing and counseling was the same between government and non-governmental respondents, except for in Asia and the Pacific and Eastern Europe and Central Asia (Table 3).

**Table 3:** Implementation of testing and existence of laws that hinder access to services as reported through the NCPI and coverage from surveys (data from most recent NCPI reporting round and most recent survey data available), including selected countries with available data

Region	Median implementation score reported through NCPI Part A	Median implementation score reported through NCPI Part B	Median % coverage testing among general population	Median % coverage testing among MSM	Median % coverage testing among SW	Median % coverage testing among PWID
Asia and the Pacific	4	3	9	42	43	35
Caribbean	4	4	19	52	65	82
East and Southern Africa	4	4	33	60	64	61
Eastern Europe and Central Asia	4	3	14	37	46	40
Latin America	3	3	18	35	53	47
Middle East and North Africa	3	3	6.6	31	20	25
West and Central Africa	3	3	12	45	68	24

The NCPI asks respondents to identify whether each of a list of HIV prevention interventions has been implemented by rating to what extent they agree that the majority of people in need have access to each intervention using the following scale: 1—strongly disagree, 2—disagree, 3—agree, 4—strongly agree, N/A

Across all regions, respondents indicated being in agreement or strong agreement that the majority of people in need had access to HIV testing and counseling, despite median testing coverage as reported through surveys being low, below 34%, among the general population and with median regional coverage ranging between 31 and 60% among men who have sex with men, 20–68% among sex workers and between 24 and 82% among people who inject drugs. Generally in regions with lower reported testing in the survey data, countries also reported a lower implementation score in the NCPI (Table 3).

### The Value of the NCPI Reporting Processes at National and Global Levels

#### Process

The NCPI reporting process has changed little since its inception. The biggest change occurred between 2004 and 2006, when the NCPI was restructured to include two sections to be completed by government and non-governmental actors respectively. Although dialogue between government and civil society has been encouraged through the NCPI from the outset, more detailed guidance has been provided since 2006, including recommended steps for dialogue between government and other partners to discuss differences between Parts A and B.

#### Reporting Rates

**Fig. 1:**
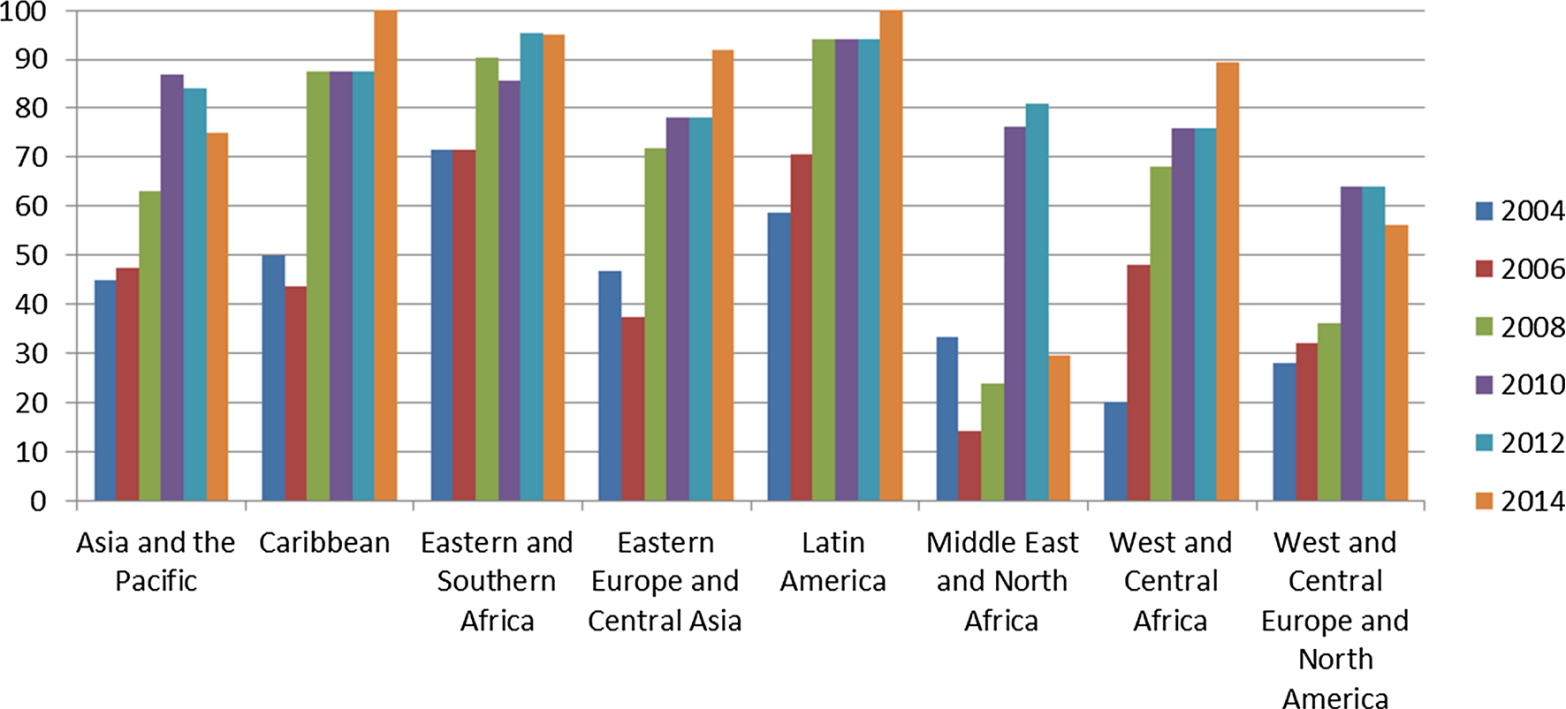
Percentage of member states per region submitting the NCPI 2004–2014

High reporting rates suggest the majority of countries are invested in the process, as reflected by the submission of both Parts A and B. There has been a steady increase in the number of countries submitting the NCPI, from its inception in 2004 when 88 countries reported, 95 in 2006, 136 in 2008, 171 in 2010, 173 in 2012 and with a slight decline in 2014 (In the 2014 reporting round 117 countries submitted the complete NCPI and 43 countries in Europe and Central Asia submitted responses to the Dublin Declaration questionnaire,^3^ which includes a sub-set of NCPI questions) (Fig. 1). Countries have the option to indicate in the GARPR online reporting tool whether an indicator, including the NCPI, is not relevant or whether data are not available.

#### Engaging Non-governmental Actors in Reporting

By including some of the same questions in both parts of the questionnaire, the NCPI allows for government and non-governmental partners to learn from one another in the information available to them. In this regard, the NCPI differs from traditional monitoring and reporting processes by providing a space for non-state actors to actively engage and provide confirmation or alternative perspectives. It is expected that there will be differences of opinion.

A comparative analysis of responses on the existence of non-discrimination laws from Parts A and B in 2012 and 2014 provides insight on the value in having both questionnaire parts. Consistently across regions there are differences in the number of countries reporting the existence of a general law (not specific to HIV) on non-discrimination by governments and non-governmental partners. In Asia and the Pacific, East and Southern Africa and Latin America the number of countries reporting such a law was higher from non-governmental partners than governments in both rounds.

Differences were also observed in the majority of regions between responses from government and non-governmental partners on the existence of laws that present obstacles to effective HIV prevention, treatment, care and support for men who have sex with men and sex workers (Table 2), an important issue for dialogue and negotiation.

#### Encouraging and Enabling Multistakeholder Dialogue

Respondents to the 2014 NCPI survey identified the tool as most meaningful in generating dialogue between civil society and government, and measuring progress in development and implementation of national HIV policies, strategies and laws. The value placed on dialogue generated through the NCPI is noted by respondents, specifically promoting transparency in reporting, engaging broader government institutions in reporting, promoting public dialogue about HIV policies and legislation and engaging with civil society and government are key areas in which the NCPI has been reported as meaningful by approximately one-third of survey respondents (Table 4).

**Table 4:** Areas where the NCPI has been most meaningful (from responses to a 2014 survey on a review of the NCPI)

Purpose	Percent (of 280 total responses) of survey respondents reporting the NCPI has been most meaningful in each area (%)
Engaging with civil society/government	43
Measuring progress in the implementation of national HIV policies, strategies and laws	40
Measuring progress in the development of national HIV policies, strategies and laws	39
Validating the national report	32
Promoting public dialogue about HIV policies and legislation	30
Engaging broader government institutions in the reporting process	30
Promoting transparency in reporting	28

#### NCPI Data Use

Based on the 2014 NCPI survey, use of NCPI data once made publicly available differed between respondent groups, with the greatest reported use among those from the UN and National AIDS Programs and the lowest use among civil society representatives.

Respondents stressed that the NCPI should collect objective and evidence-based information to serve not only measurement but also advocacy. Of survey respondents who reported not using the data, 25% indicated they did not trust its quality or did not consider it representative, 23% had difficulties accessing it, and 41% indicated that the data collected did not respond to their needs. Other reasons cited for not using the NCPI data included that other systems to collect policy-related data were available at national level and therefore the NCPI was essentially a report prepared for the UN, although the dialogue between actors generated by completing the tool was reported as useful [18].

Several respondents suggested that more support and guidance for the analysis of data collected and development of a plan to use results could be useful. In terms of data collection, shortening the questionnaire was noted as a potential improvement for any future iteration, improving the translations of the questionnaire, and making the tool more user-friendly to complete and submit. The possibility of adapting the questionnaire to reflect different epidemic contexts was also proposed.

#### Perceived Utility of the NCPI

The majority of respondents to the 2014 NCPI survey indicated the NCPI has been useful and that it, or a similar tool, continues to be relevant (75% of respondents answered yes, and 20% indicated it is partially relevant).

## Discussion

What lessons can be drawn from the NCPI which, to our knowledge, is the first and only effort to collect data systematically on country self-reported national laws and policies related to HIV?

### The Purpose of the NCPI

The NCPI has evolved into a comprehensive questionnaire collecting information on laws, policies and regulations related to HIV, as well as other aspects of program implementation. Reporting rates remain high.

Initially it was envisioned that data collected through the NCPI would be analyzed through the construction of an index which could provide insight into a country’s AIDS-related policy environment. However, it was found challenging to construct a meaningful index that adequately reflected the breadth and heterogeneity of available data. Although indices have been recognized as providing an easy-to-understand measure of complex issues and effective tools to support advocacy efforts, they risk oversimplifying and overstretching available data [19]. The NCPI has therefore transitioned into an instrument, with analysis performed on the basis of individual questions—each of which measures some aspect of whether a country provides an enabling environment for a robust AIDS response.

### Information that can be Meaningfully Captured Through the Instrument

The responses captured through the NCPI sometimes differ from data reported through other sources, such as the Law Database or population-based surveys. For example, the Law Database only includes information about a specific set of legal obstacles for effective HIV prevention, treatment, care and support, e.g. criminalizing same-sex-relationships for MSM. The NCPI, by contrast, asks more generally if the country has laws, regulations or policies that present obstacles to effective HIV prevention, treatment, care and support for key populations and vulnerable groups. In other words, the NCPI recognizes that access may be restricted by measures and practices beyond criminal law. This can explain why, for example, the UNAIDS Law Database and the responses from civil society in Latin America differed from one another on reported obstacles (men who have sex with men in a third of the countries, and in more than half of all countries for sex workers, even where no criminal law is recorded) (Table 2). Conversely, in a country where laws exist which criminalize same sex relationships and government and/or non-governmental actors answer in the NCPI that there are ‘no obstacles’ –a lack of understanding or willingness to acknowledge that such obstacles exist is revealed. This can be seen in many regions, but most notably in West and Central Africa (Table 2). Discussions on legal obstacles for key populations are therefore strongest if findings from both the NCPI and complementary validated sources are consulted.

There are also differences in relation to service coverage as reported through surveys and the implementation score reported through the NCPI. Such variation may arise through the way the measures capture coverage, with one focusing predominantly on access and the other on use. The NCPI asks if “The majority of people in need have access to…” whereas most surveys seek to understand if people actually have received services, in this case an HIV test in the last 12 months.

The criteria which respondents take into consideration in responding to the NCPI raise another set of issues. Respondents rarely document the reason for the answer provided. This makes interpretation difficult and at times has led to suggestions of unreliability, as responses do not reflect data available through validated (e.g. quantitative) methods. The subjective interpretation of questions by respondents as well as the lack of clarity of some terminology and definitions [20] may hinder the possibility of comparison over time for a country, as well as across countries and therefore global monitoring. In some cases, responses may reflect perceptions of implementation of laws or policies, for example, rather than their actual implementation which is in and of itself important, particularly as concerns key populations.

As a starting point for a discussion at country and global levels, the NCPI process has proved useful. Going forward, in relation to its programmatic components the Instrument’s greater utility may be achieved by focusing questions related to programmatic elements on areas that can provide further insight on the reasons for good or poor performance, or related qualitative aspects that quantitative indicators cannot capture.

### The Value of the NCPI

High reporting rates suggest that the majority of countries continue to consider monitoring the policy and legal environment of the AIDS response relevant to improving access and use of services—or complying with international norms [33]. Limited use of NCPI data once collected may be due to challenges in interpreting responses meaningfully [21], as well as challenges in accessing the data in a format that facilitates broad dissemination, analysis and use. Making NCPI data publicly available in an easily analyzable format, rather than simply publishing reports on the UNAIDS webpage in PDF format, could increase its use. A database of responses is maintained by UNAIDS and used to produce analyses of annual responses and trends for consistently-reporting countries, along with extracts of narrative responses. Tabulations of NCPI data can be provided by UNAIDS upon request.

As described above, the differences between government and non-governmental responses have been extremely useful for triggering dialogue among country stakeholders [22]. Variances in responses between Parts A and B suggest there is value in providing a space for government and non-governmental actors to report separately, and subsequently engage in dialogue around identified differences.

Perceptions of the existence of laws or policies, even if not accurate, may impact service delivery and uptake and is therefore important country level information. The broad and open questions of the NCPI on the existence of obstacles may also facilitate awareness of the legal environment more broadly. Probing the existence of specific laws and policies may be something captured through other existing tools (such as the above-mentioned Law Database), and may not be a good use of limited time and resources. However not including questions on the existence of laws and policies provides less scope for addressing specific harmful laws and policies, unless additional details from other sources are provided and discussed during stakeholder dialogues or narrative responses.

Additional data on the actual implementation of laws and policies, or related practices, would be an important addition to any future iteration of the tool [21]. Modifications to the questionnaire content should also account for the evolution in data availability on HIV-related laws and policies from other sources..

Policy processes are notoriously complicated, the term itself is contested, and there is often a major disconnect between stated policy and what gets implemented in practice [23]. As a participatory policy monitoring tool, the NCPI could be well placed to capture gaps between law and policy existence and implementation, which can have an important impact on access to services. Moreover, some of the concepts which the NCPI attempts to capture, while elusive and difficult to measure, are important and not well captured through other mechanisms. For example, the concepts of ‘political will’ and ‘leadership’—one knows when they are present, but to capture them through the number of times reference is made to an issue in leader’s speeches, or in party manifestos, may or may not provide an adequate measure of ‘will’. President Mbeki of South Africa exercised significant political will in relation to his ‘denialist’ position on AIDS—but was at odds with the need for evidence-informed prevention and treatment services [24].

The space for narrative responses in the NCPI provides important insights for the interpretation of reported data; even as the human resource requirements for their analysis have been cited as challenges to the greater use of such information [18]. By documenting progress in the development and implementation of national HIV policies, strategies and laws, which may not be captured elsewhere, these narratives provide important indications of progress, particularly for laws or policies that may take a long time to change, such as abolishing a law that criminalizes a behavior.

The NCPI process has created an important space for dialogue between governments, civil society and other stakeholders on a range of policy issues that play an important role in determining access to services and a human rights-sensitive HIV response^4^ [18]. The AIDS response is marked by the engagement of people living with HIV and those affected by the epidemic. The areas where progress has been greatest have been where mutisectoral, multistakeholder engagement has been fostered as the norm, particularly that of affected communities [25]. Community participation and engagement have been found to improve access to services in various settings through the construction of citizenship, strengthening of participation practices, and strengthening the responsiveness and accountability of states, although the results of engagement have been found to also vary by context and form of participation [26]. GARPR has provided a platform for such engagement and the NCPI provides the data to begin discussion of issues across the breadth of the response. Although a national consultation or dialogue on NCPI responses is encouraged in the recommended process for completing the NCPI, these have not taken place systematically across countries or over time. Respondents to the 2014 NCPI survey noted limited time to complete the tool, in particular given its length, and lack of human and financial resources to bring stakeholders together as challenges. Limited capacity of civil society organizations to provide answers and engage with the tool was also cited. Respondents recommended a simpler and shorter questionnaire that would allow for more meaningful responses, as well as to facilitate engagement and dialogue among stakeholders. [18].

The extent to which the instrument can contribute to the reform of policies or legal frameworks which promote more inclusive, evidence-informed and human rights-based responses has not yet been assessed, and is an area ripe for investigation.

While not perfect, the NCPI has been recognized as one of the most comprehensive sources of information on HIV laws and policies, and as having contributed to the assessment of political commitment through standardized questions across countries [27]. NCPI-based questions have been integrated and adapted in monitoring and evaluation guidance and tools by partners, including The United States President’s Emergency Plan for AIDS Relief (PEPFAR) [28] and Measure Evaluation [29]. Similar tools have been developed for monitoring and evaluation of specific program areas, including certification for the virtual elimination of mother-to-child HIV (and syphilis) transmission [30], assessments of legal and policy environments related to reproductive, maternal and newborn health [31], and the International Conference on Population and Development (ICPD) [32] review process [32].

## Limitations

Limitations to this paper’s findings and analysis include: (1) trend analysis—the respondents to the NCPI in many countries have changed over time; some responses are subject to the views of the individuals, affecting on the ability to compare and interpret results over time; (2) author’s roles vis-a-vis the NCPI—the authors have a history in working on the NCPI, with the insights on the tool, its history and its use (this emic perspective is a strength but can introduce observer bias); (3) this paper relies in part on an earlier analysis of the NCPI—the analysis of that study influences this paper’s scope, selection of data sources and interpretation of findings; (4) impact analysis not available—the content of narrative responses to NCPI questions, and the influence of NCPI on policy changes have not been studied, but could provide further insights on the scope and utility of the Instrument.

## Conclusion

Ten years of NCPI experience have demonstrated the importance of the NCPI in describing the political, policy and legal environments for national HIV responses, for programmatic reviews and to stimulate dialogue among stakeholders. Our analysis suggests the tool can be further strengthened and needs to be complemented by more objective quantitative data, accounting for data sources not previously available. Similar tools and processes could serve to monitor and report on the policy and legal frameworks governing the determinants of vulnerability and risk to the range of health issues receiving attention in the implementation of the 2030 Agenda for Sustainable Development.

In relation to the NCPI, as the epidemic and response evolves, we conclude that the NCPI needs to be updated in five areas. First, it will need to evolve to measure new policies and issues in the AIDS response, such as in relation to access for more recently acknowledged key populations (e.g. transgender people and prisoners), viral load monitoring, discrimination faced in health care settings, the integration of services, access to justice and Universal Health Coverage for people living with HIV, among others. Second, there is a need to update the tool to capture data on policy implementation and quality aspects of the response, beyond reporting on the existence of laws and policies, while focusing on relevant information on laws and the policy environment not captured elsewhere and around which it would be of value to have dialogue among national stakeholders. Third, a better set of instructions (providing clarity of some terminology and definitions) should be added and questions made more specific, to limit the subjectivity of responses. Fourth, there is greater potential for wider use of the findings, which might be facilitated by better guidance on possible analyses including triangulation. Finally, given that the greatest value added of the NCPI process appears to be the platform it provides for multistakeholder and multisectoral dialogue, revisions to the NCPI, or any future tools, should include a stronger framework for civil society roles within monitoring and reporting processes. Finding the right balance between keeping such an instrument as concise as possible and also taking these five proposals into account will require trade-offs and further thought.

Similar tools and processes may be of interest/utility in other areas of global health and development (e.g. laws regarding age of consent for testing and accessing health services, sexual activity, condom accessibility; women’s rights including property rights, inheritance, and custody; sexual and physical violence including within marriage; access to sexual and reproductive health and rights; child marriage; wrongful arrest and coercion). Our analysis suggests that the NCPI experience is relevant to the SDGs, particularly its inclusive process of monitoring and reporting [33] and its focus on the structural determinants of vulnerability and risk. An analysis of the health targets of the 2030 Agenda for Sustainable Development concluded that successful implementation will likely hinge on a number of major reforms in global health including more inclusive, multistakeholder, multisector governance approaches and new means of regulating and legislating upstream determinants [34]. In the same way that the AIDS response needs laws, policies and regulations governing non-discrimination [2], the global health agenda will require attention to non-discrimination as well as a raft of new measures including those governing the regulation of commercial sector drivers of risk and exposure to non-communicable diseases, environmental pollutants, among others [35]. It is arguably the case that the NCPI approach would be relevant to participatory monitoring of targets in the health and other goals of the SDG framework. We argue that the NCPI suggests a way forward to promote and support human rights, leaving no one behind and accountability in implementing Agenda 2030.

## Footnotes

1 Data in the law database is a compilation of data collected and maintained by UNAIDS through reviews of available documents. It is based on a compilation developed with partners in 2010. It is referenced as an indicative source that may not be comprehensive and is not validated by countries.

2 The objectives of the NCPI review were to assess the tool’s purpose in the post-2015 environment and propose a new tool towards 2020/2030.

3 For more information on the Dublin Declaration, please see http://ecdc.europa.eu/en/activities/diseaseprogrammes/hash/Pages/monitoring-dublin-declaration.aspx.

4 A mechanism of “shadow reporting” was developed for situations in which civil society did not feel adequately involved in reporting. For further discussion of this modality, please see Smith et al. “The Role of Civil Society Organizations in Monitoring the Global AIDS Response” in this supplement.
